# P53 Gene as a Promising Biomarker and Potential Target for the Early Diagnosis of Reproductive Cancers

**DOI:** 10.7759/cureus.60125

**Published:** 2024-05-11

**Authors:** Aswathi R K, Suresh Arumugam, Natrajan Muninathan, Kuppusamy Baskar, Deepthi S, Dinesh Roy D

**Affiliations:** 1 Medical Biochemistry, Meenakshi Academy of Higher Education and Research, Chennai, IND; 2 Central Research Laboratory, Meenakshi Medical College Hospital and Research Institute, Kanchipuram, IND; 3 Research and Development, Meenakshi Academy of Higher Education and Research, Chennai, IND; 4 Centre for Advanced Genetic Studies, Genetika, Thiruvananthapuram, IND

**Keywords:** tumor suppressor gene, reproductive cancer, biomarker, p53 gene, cancer

## Abstract

One of the crucial aspects of cancer research is diagnosis with specificity and accuracy. Early cancer detection mostly helps make appropriate decisions regarding treatment and metastasis. The well-studied transcription factor tumor suppressor protein p53 is essential for maintaining genetic integrity. p53 is a key tumor suppressor that recognizes the carcinogenic biological pathways and eradicates them by apoptosis. A wide range of carcinomas, especially gynecological such as ovarian, cervical, and endometrial cancers, frequently undergo *TP53* gene mutations. This study evaluates the potential of the *p53* gene as a biological marker for the diagnosis of reproductive system neoplasms. Immunohistochemistry of p53 is rapid, easy to accomplish, cost-effective, and preferred by pathologists as a surrogate for the analysis of *TP53* mutation. Thus, this review lays a groundwork for future efforts to develop techniques using p53 for the early diagnosis of cancer.

## Introduction and background

Cancer is a multifaceted, intricate illness that can affect any region of the body. Uncontrollably growing aberrant cells that can infiltrate adjoining tissues and spread to other organs are its defining feature. If successive variations occur in the genes that control normal cell division and proliferation, these mutations can accumulate over time and contribute to tumorigenesis. Cancer is the second most common cause of mortality globally and its prevalence has risen in recent decades [1]. A series of recurrent gene mutations that alter cell functions produce a disruption in the cell cycle, which results in aberrant proliferation. Essential genes become dysfunctional as a result of the disruption of cellular connections [2]. The accumulation of these genetic changes over time causes abnormal growth and division of cells, forming tumors and invading surrounding tissues. Numerous variables, such as lifestyle decisions, environmental exposures, and genetic predispositions, might cause these mutations. Cancer is a worldwide health issue affecting millions of individuals, despite advancements in medical research and treatment choices.

According to the 2020 global data, putting aside non-melanoma skin cancer, lung cancer accounted for 15.4% of all newly diagnosed instances of cancer in men globally making it a highly prevalent cancer type in this demographic. Apart from non-melanoma skin cancer, three cancers, namely, lung, prostate, and colorectal, accounted for 41.9% of all cancer cases. Among the other common cancers that contributed more than 5% were those of the stomach and liver. Breast cancer accounted for 25.8% of all recently reported malignancies in women globally, being the most common cancer type in women overall. Other than non-melanoma skin cancer, three cancers, namely, breast, colorectal, and lung malignancies, accounted for 44.5% of all cancer cases in women. Cervical cancer accounted for 6.9% of all recent cases in 2020, making it the fourth prevailing malignancy in women. While cervical, endometrial (4.8%), and ovarian (3.6%) cancers are common and contribute to significant cancer morbidity and mortality throughout the world, vulvar (0.5%) and vaginal (0.2%) cancers are incredibly rare in the female reproductive system [3].

Cancer diagnosis cannot be made with a single test. In addition to other laboratory testing, imaging tests, and biopsies are frequently utilized. MRI, CT scan, positron emission tomography (PET) scan, nuclear scan, bone scan, ultrasound, X-rays, mammography, and biopsy are common imaging procedures used in its diagnosis. Mammography is unable to pick up on tiny lesions that MRI can identify. On the other hand, its low specificity and high cost are disadvantages [4]. Biomarkers are helpful in diagnosis, disease monitoring, predicting recurrence, and evaluating therapy efficacy. Low levels of cancer markers are associated with inadequate early detection [5].

The tumor suppressor *P53* gene expression represents the information complementary to the patient’s prognosis, survival, and morphology [6]. Loss of p53 function has been related to the widespread presence of *TP53* mutations in a variety of human malignancies that are essential for the growth of cancers [7]. Limited studies have shown that *p53* mutation is absent in a few cases. However, the majority of cancers do contain *TP53* mutation. Hence, for diagnostic purposes, the identification of alterations in *TP53* can be a helpful indicator. Realizing the importance of the *TP53* mutation discussed above, we have investigated the gene at the molecular level with an emphasis on identifying cancers precisely and affordably and attaining disease treatment in its early stages.

## Review

*p53*: the guardian of the genome

**Figure 1:**
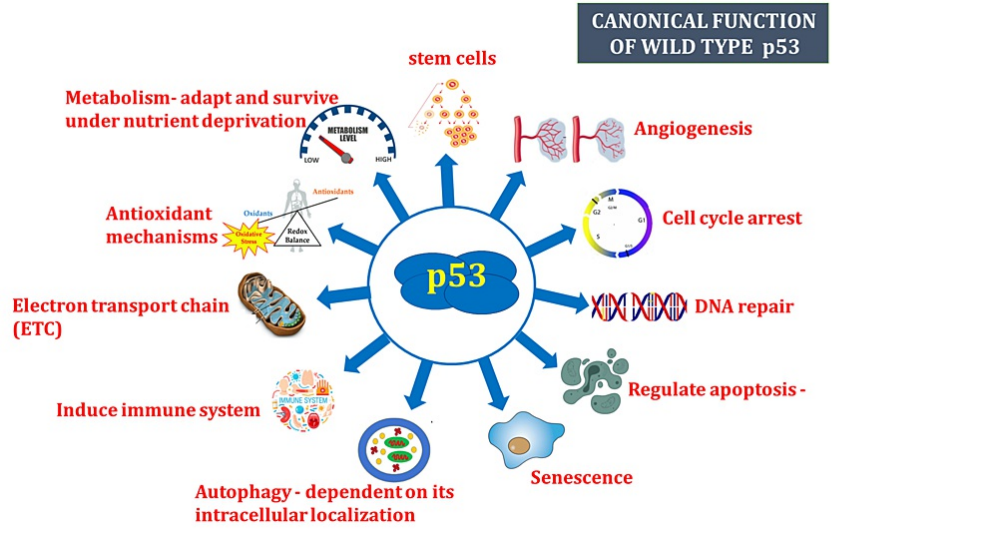
Canonical functions of wild-type p53. The functional properties of p53 are depicted in the figure. The *p53* gene regulates a wide range of physiological functions, including immunological response, stem cell maintenance, cell metabolism, mitochondrial respiration, autophagy, angiogenesis, senescence, apoptosis, and fertility. It also delineates the regulatory mechanisms by which these activities are accomplished, including physical interactions with binding partners and transcriptional modification of target gene expression [8-16]. Image credit: Aswathi Ramachandran.

The *p53* gene is a transcriptional regulator located in the nucleus that controls many different cellular processes (Figure 1) (Table 1). To maintain homeostasis and genomic integrity, numerous target genes are regulated by *p53*, which binds to DNA through proximal promoters and distal enhancers. By preventing the growth of cancer cells or eradicating them, *p53* has emerged as a crucial impediment to the progress of cancer [8]. Beyond that, *p53* contributes to the differentiation of stem cells, aging, metabolism, DNA damage response, and fertility. It modulates several genes that are involved in cellular senescence, repairing of DNA, apoptosis, and arresting of the cell cycle. *p53* is essential for promoting DNA repair because it slows down the cell replication cycle, which provides more time for the repair mechanisms to stabilize the genome [9]. The role of *p53* as a tumor suppressor in malignancies has long been a subject of interest, which has earned it the moniker “guardian of the genome” [10].

**Table 1:** Canonical functions of wild-type p53 [8-16]. CDKs = cyclin-dependent kinase; cdc2 = gene for cyclin-dependent protein kinase Cdk1/Cdc2; GADD45 = growth arrest and DNA damage-inducible 45; PUMA = p53 upregulated modulator of apoptosis; Bax = Bcl-2-associated X protein; Bak = Bcl-2 antagonist killer 1; TRIAP1 = TP53 regulated inhibitor of apoptosis 1; Bcl-xL = B-cell lymphoma-extra-large; MHC = major histocompatibility complex; GLS2 = gene codes for glutaminase 2; TIGAR = TP53-induced glycolysis and apoptosis regulator Table credit: Aswathi Ramachandran.

Cellular functions	Role of p53 protein
Cell cycle arrest	G1: S phase - enhances the production of p21 protein which inhibits CDKs. G2: M phase - inhibits Cdc2 by the production of GADD45, p21, and 14-3-3 sigma
DNA repair	Enhances the production of damage-specific DNA-binding protein 2 and XPC complex subunit (DNA damage recognition and repair factor)
Regulate apoptosis	In both transcription-dependent and independent manner. Apoptosis and cell survival depend on the members of the BCL-2 family. Induction pro-apoptotic BCL-2 family members → PUMA, BAX, BAK, NOXA → promotes apoptosis. Induction of anti-apoptotic BCL-2 family members → BCL-2, BCL-XL, TRIAP1 → inhibits apoptosis
Senescence	Induces transcriptional activation of the CDK inhibitors, p21 and p16 → senescence, associated with nuclear deformation, Lamin A/C expression, and p16 induction
Autophagy	Depends on its intracellular localization. Under cellular stress, P53 translocating to the nucleus → activates autophagy. Under normal physiological state, cytoplasmic P53 → inhibits autophagy
Induce immune system	Represses pro-inﬂammatory responses. Represses NF-kB → promotes an anti-inﬂammatory microenvironment. Upregulates MHC-I → promotes T-cell recognition
Electron transport chain (ETC)	Synthesis of cytochrome c oxidase 2, required for mitochondrial cytochrome c oxidase assembly, regulating the normal functioning of ETC
Antioxidant mechanisms	Increases levels of GST (glutathione S-transferase) to avoid deleterious eﬀects of ROS
Metabolism	Adapts and survives under nutrient deprivation. Carbohydrate metabolism: Represses the expression of various glucose transporters and activating TIGAR → downregulates glycolysis. Regulates gluconeogenesis and pentose phosphate pathway. Lipid metabolism: enhances fatty acid oxidation and inhibits fatty acid synthesis. Suppresses the mevalonate pathway → inhibits the biosynthesis of cholesterol and nonsterol isoprenoids. Amino acid metabolism: transcriptional regulation of GLS2 → replenishes TCA intermediates
Stem cells	Regulates stem cell proliferation, differentiation, maintenance of stem cell genetic stability.
Angiogenesis	Limits angiogenesis by interfering with central regulators of hypoxia. Inhibits the production of proangiogenic factors and vascular endothelial growth factor (VEGF). Increases the production of endogenous angiogenesis inhibitors

The principal defenses against the formation of tumors are regulated by the transcription of genes, regulated by the p53 protein when it attaches to a particular response region in DNA. If DNA damage is found to be permanent, the p53 protein triggers apoptosis or cell senescence [11]. Increased p53 levels can induce growth arrest, senescence, or apoptosis; however, the effects vary depending on the circumstances. These outcomes are accepted as mechanisms for suppressing tumors, as they prevent the uncontrolled proliferation of transformed cells and may even eliminate them entirely [12].

Through its transcription-independent function, p53 is also able to regulate the mitochondria’s ability to initiate apoptosis [13]. The ability of non-mutant *p53* to aid the cell in adapting to and surviving minimal stress circumstances, such as oxidative stress and metabolic stress, is one of its tumor-suppressing properties [14]. p53 is capable of triggering autophagy, which has been implicated in cancer suppression, along with its functions in inducing growth arrest, senescence, and apoptosis [15]. p53 also modulates a variety of metabolic pathways, including those related to glucose, lipid, and amino acid metabolism. Because of this, p53 helps a cell adapt to and survive minor metabolic stress [16].

p53: a fencekeeper for the growth and proliferation of cells

**Figure 2:**
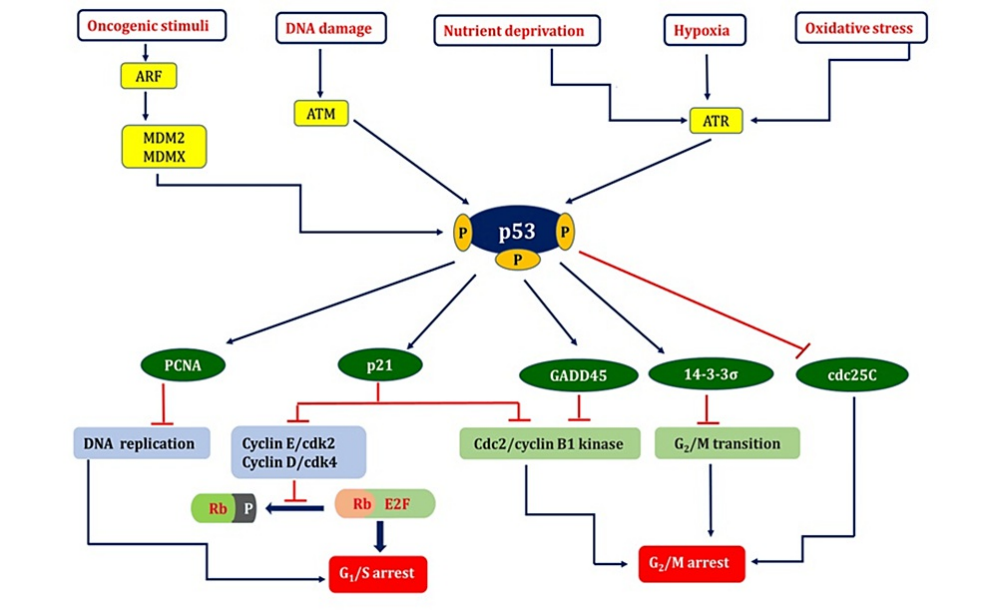
p53: a fencekeeper for cell growth and proliferation. Many cellular stress cues trigger the activation of *p53*. *p53* activators include hypoxia, oxidative stress, oncogene activation, nutritional stress, and DNA damage. Activation of *p53* induces the transcriptional upregulation of one or more target genes, which mediate the biological consequences depicted in the figure, and lead to cell cycle arrest at the G1/S or G2/M checkpoints [17-21]. ARF = alternative reading frame (tumor suppressor protein); MDM2 = murine double minute 2; MDMX = murine double minute X; ATM = ataxia telangiectasia mutated; ATR = ATM-related; PCNA = proliferating cell nuclear antigen; GADD45 = growth arrest and DNA damage-inducible 45; cdc = cell division cycle 25C; Rb = retinoblastoma protein; P = phosphate; E2F = eukaryotic transcription factors; CDK = cyclin-dependent kinase; ┴ = inhibit; ↓= activating Image credit: Aswathi Ramachandran.

p53 can initiate several physiological responses and can be triggered by several stimuli, including DNA damage caused by ionizing irradiations such as ultraviolet, X-ray, and gamma rays, ribosomal or nucleolar stress, mitogenic signaling, improper proto-oncogene activation, and hypoxia. These include halting the cell cycle, repairing damaged DNA, and inducing specific reactions causing cell destruction or metabolic modifications within the cell [17]. A vast array of genes involved in many processes is triggered to express themselves when p53 is activated. Extensive genetic studies have identified the most well-known p53-focused genes that are implicated in arresting cell division (Figure 2) and programmed cell death in response to DNA damage.

The transcriptional stimulation of p21/WAF1 is predominantly caused by phosphorylated p53. The cell replicating cycle halts during the G1-S transition phase when p21 interacts with the cyclin D/Cdk4 and cyclin E/Cdk2 complexes. The suppression of cyclin-dependent kinase 2 and cyclin-dependent kinase 4 by p21 prevents pRb from being phosphorylated and increases pRb binding to E2F1, which, in turn, encourages transcription suppressing of E2F1 targets, which is essential for DNA duplication and the advancement of the cell cycle [18]. The potent connection between the C-terminus region of p21WAF1 and the interdomain connecting loop of PCNA may halt DNA replication in vitro, which is one of the factors that contribute to its cell-cycle arrest properties [19].

Research has demonstrated that p53 can impede the cdc25C promoter and target its transcriptional suppression, resulting in cell cycle arrest during the G2-M transition phase when DNA mutations occur [20]. The completion of the G2-M transition depends on the nucleus localization of the nuclear cyclin B1 protein. Activated GADD45, a p53-regulated DNA damage-inducible protein, reduces Cdc2 kinase activity and inhibits cell proliferation. The reduced level of nuclear cyclin B1 correlates with the reduction of Cdc2/cyclin B1 kinase activity [21].

The p53-MDM2 feedback loop

Although it is often found in a dormant state, the p53 growth silencer functions as the “gatekeeper of the genome” when DNA damage occurs. In response to genotoxic stimuli, p53 initiates objective properties that start the pathways leading to the halting of the cell cycle, senescence, programmed cell death, and preventing DNA damage from progressing to heritable alterations [22]. Different types of inhibitors regulate p53 migration after DNA damage compensation. The main function of MDM2 is to effectively inhibit p53 and prevent undesirable cell cycle arrest or cell death when conditions are normal. A gap between p53 actuation and MDM2 development characterizes the dynamic phase of p53. p53 initiates MDM2 quality articulation in an autoregulatory circle [23].

**Figure 3:**
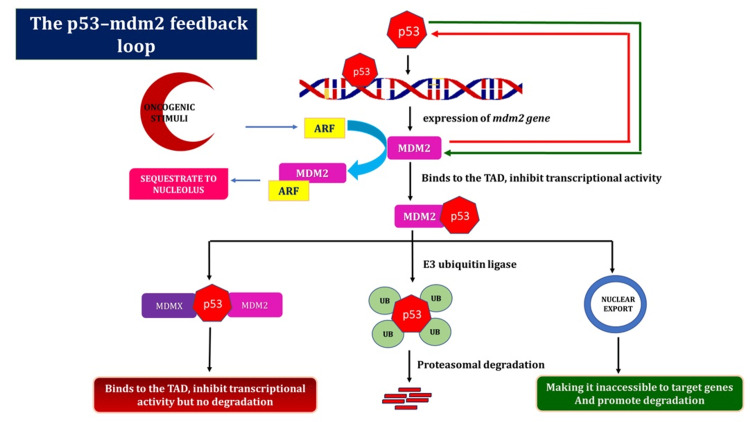
The autoregulatory feedback loop between MDM2 and p53. An abundance of correlative details indicates that the primary mechanism of p53 regulation in both normal and stressful circumstances is the p53-MDM2 autoregulatory loop. This diagram showed how MDM2 emerged as p53’s primary negative regulator. As MDM2 is a transcriptional target of p53, a negative feedback loop is created. Following the stabilization of the damage, active p53 attaches to the MDM2 P2 promoter and stimulates transcription of the gene. MDM2, in turn, disables p53 via one of the three mechanisms [22-25]. ARF = alternative reading frame (tumor suppressor protein); MDM2 = murine double minute 2; MDMX = murine double minute X; TAD = transactivation domains; UB = ubiquitin Image credit: Aswathi Ramachandran.

To prevent needless cell cycle arrest or death, MDM2 represses p53 under non-stressful conditions. The delay that occurs before MDM2 develops directs p53’s action, and p53 then upregulates MDM2 in a negative feedback loop [24]. As per the overall perspective, MDM2 employs three tools to suppress p53 (Figure 3). Initially, it attaches to the N-terminus transactivation region in p53, impeding its transcriptional function. Second, MDM2 directs ubiquitination and the proteasome to degrade p53. Finally, MDM2 collaborates with p53 to expel it from the core and prevent it from binding to transcriptional co-activators. A similar explanation states that MDMX essentially prevents p53 from functioning by joining its transactivation site [25].

Mutation in the *TP53* gene

A gene called *TP53*, which codes for the protein p53, is most commonly altered in human malignancies [26] (Table 2). One important function of this protein is to stop tumors from growing and developing. On the other hand, research has revealed that mutations in the *TP53* gene cause malfunctioning p53 protein in over half of all types of carcinomas in humans [27]. The *p53* tumor suppressor gene mutation has been linked to malignant transformation. A substantial number of *TP53* alterations that develop during the progression of cancer are missense mutations which lie within the region of the gene encoding the location-specific, DNA-binding domain, supporting the notion that DNA binding and transcription are essential for tumor suppression [28].

**Table 2:** Frequency of p53 gene mutation corresponding to different types of cancer. Table sources referred from [29].

Types of cancer	Frequency of mutant p53 (%)
Diffuse glioma	48.34
Head and neck squamous cell carcinoma	68.26
Non-small-cell lung cancer	66.48
Esophageal squamous cell carcinoma	93.68
Invasive breast carcinoma	32.66
Esophagogastric adenocarcinoma	54.09
Hepatocellular carcinoma	31.17
Colorectal adenocarcinoma	53.03
Pancreatic adenocarcinoma	58.7
Endometrial carcinoma	41.98
Ovarian epithelial tumor	64.55
Bladder urothelial carcinoma	50.36
Sarcoma	46.67

An unusual protein that is translated as a result of *TP53* gene mutations has significance in upsetting the molecular functions associated with p53. These mutations exhibit trans-dominant suppression in comparison to their wild-type counterparts, losing their ability to suppress cancer growth [30]. Mutant p53 proteins may be considered to be oncogenes that encourage carcinogenesis, with the degree to which their neomorphic action varying based on the specific *p53* mutation [31].

*TP53* is unique compared to other tumor suppressor genes in that it can be subject to a wide range of missense mutations, which lead to a variety of p53 proteins with varying degrees of functional diversity. Mutations in *p53*, homo-tetrameric transcriptional factor, result in dominant-negative (DN), gain-of-function (GOF), and loss-of-function (LOF) phenotypes. Common p53 non-synonymous mutations not only result in a GOF that accelerates the growth and spread of malignancies but are also unable to stop the growth of tumors [32]. Additionally, Li-Fraumeni syndrome (LFS), a rare strongly inherited malignancy, is caused by germline *TP53* mutations [33].

The disclosure of a heritable cancer predisposition is considered to be an effective strategy in preventing the development of cancer and associated deaths, as it allows for early identification and risk management. To date, though, there is only one proven method to dramatically reduce the chance of developing malignancy and dying from it: disclosing one’s *TP53* carrier status [34]. Approximately 70% of *TP53* variants associated with cancer development are non-synonymous substitutions. These mutations are thought to be extremely permeable and harmful, and they are usually found in the core DNA-binding region. However, the degree of penetrance may fluctuate concerning a particular kind of mutation. For instance, p.R213Q has a minimal penetrance for the development of cancer, whereas the p.R248W *TP53* variation has a significantly greater penetrance and causes malignancies of the breast, ovaries, and colon. Recent studies have suggested that there are 10 times more *TP53* mutations with potential for disease in the community than was previously thought. Furthermore, the degree of penetrance might vary more than is currently thought, underscoring the necessity of further research to fully comprehend the complexity of cancer risk syndromes [35].

LFS is a rare inherited disorder linked to *TP53* gene mutations, significantly increasing the risk for diverse malignancies such as breast, brain, bone, and soft tissue sarcomas. Cowden’s syndrome is a rare hereditary genetic disorder that raises an individual’s chances of developing several malignancies, especially breast, thyroid, and endometrial cancers associated with mutations in the *PTEN* gene. Both LFS and Cowden’s syndrome have a high degree of penetrance. In contrast to the general population, this indicates that those who inherit a mutation in one of the aforementioned genes have a significantly increased lifetime chance of acquiring cancer [36].

*TP53* variant effects differ between genders; for extremely permeating alterations in the DNA-binding domain, women experience a nearly 100% lifetime cancer risk, whereas men have a 73% risk. Before age 30, these risks are 49% for females and 21% for males. Childhood cancer risk is high, with 15% of individuals affected, with 12% of females and 19% of males developing cancer [37].

The penetrance of *TP53* gene mutations may alter significantly depending on the exact mutation type, with missense mutations in the DNA-binding domain being the most pathogenic and highly penetrant. Due to abnormalities in the *TP53* and *PTEN* genes, respectively, people with LFS or Cowden’s syndrome, both uncommon hereditary cancer syndromes, are more suspectable for malignancies. Early identification and risk management through genetic testing and counseling are crucial for individuals with these genetic predispositions to effectively prevent or manage their cancer risk. Ongoing studies are required to comprehend the situation better to understand the complexities of cancer predisposition syndromes and develop more effective strategies for preventing cancer development and related deaths in individuals with inherited cancer susceptibility.

Mutant p53 as a biomarker for cancer

*TP53* gene mutation can be detected by several procedures. Blood tests for the presence of p53 antibodies are commonly performed using the enzyme-linked immunosorbent assay (ELISA). Autoantibodies (AAbs) can form as a result of modifications to tumor antigens, whether brought about by mutation, an excessive expression, or abnormal disintegration [38]. Because *p53* is mutated in distinct types of cancers, p53-AAb is an interesting biomarker [39]. Based on ELISA analysis of serum samples, it was noted two smokers had anti-p53 antibodies before a formal cancer diagnosis [40]. Potential indicators for the early detection and/or prognosis of cancer could be these antibodies’ lengthy half-lives and in vitro stability [41].

There are advantages in using immunohistochemical (IHC) staining for p53 to identify cancers with *TP53* mutations that have a poor prognosis because it has been demonstrated to correlate with the existence of missense mutations in *TP53* in a variety of tumor types. This method allows for the detection of *TP53* mutations at the protein level, providing valuable information about the functional status of the tumor suppressor gene. Moreover, IHC staining allows for the evaluation of p53 expression patterns, which could reveal information about the molecular mechanisms underlying *TP53* mutations and their role in tumorigenesis. Investigators and medical professionals can more effectively customize treatment plans for individuals with *TP53*-mutated malignancies by comprehending these patterns. IHC analysis is a popular technique that can be applied in a variety of scenarios. It not only aids in the differentiation of cells but also aids in the characterization of a tumor’s primary site. Additionally, IHC staining can serve as a valuable tool in determining prognostic factors and detecting metastases. Moreover, it can be utilized as an indicator of the response to targeted therapy. As highlighted earlier on the utility of IHC staining in evaluating p53 expression patterns and tailoring treatment strategies for *TP53*-mutated cancers, it is clear that IHC analysis is a valuable tool in cancer research. Not only can it be employed to differentiate cells and characterize a tumor’s primary site but can also serve as an indicator of the response to targeted therapy.

In addition, missense mutations were proven to be correlated with strong, diffused IHC staining for p53; in contrast, nonsense or frameshift mutations were linked with a complete absence of staining (null pattern) from p53. In the identification of invasive carcinoma, p53 IHC is now a commonly utilized diagnostic technique [42]. As most cases exhibit a p53 wild-type staining pattern, this technique is frequently applied in clinical settings [43]. However, 46% of cases have focally high p53 staining, which is consistent with subclonal *TP53* mutations [44]. p53 analysis is the only IHC marker reliable in the diagnosis and classification of cancers [45].

Perhaps the most accurate way for determining the presence of *p53* mutations is direct sequence analysis. Sequence analysis and IHC are compared in studies, and differences between the two techniques are discovered. p53 immunostaining can occasionally be seen in the absence of sequence alterations, and IHC does not always detect all *p53* mutations [46]. Owing to the development of massively parallel sequencing and analyses of large data sets using the Cancer Genome Atlas, *TP53* variations were successfully discovered, and the average incidence is over 96%. *TP53* sequence profiling is costly, lengthy, and susceptible to contamination from normal cells. Even so, yeast functional assay can also be used as an alternative technique to utilize *TP53* gene sequencing [47]. The diagnostic utility of p53 in various aspects of reproductive cancer in both males and females is reviewed below.

Prostate cancer

The prostate, urinary bladder, colon and rectum, and lungs together account for the highest incidence of cancers in men. Prostate cancer ranks fifth globally in terms of causes of mortality and is categorized as the second most prevalent cancer among men. Prostate cancer incidence and fatality rates are highly connected with age, with men over 65 having the highest incidence. An estimated 20% of prostate cancer sufferers have a family background of the disease, which can result from shared exposure to some environmental carcinogens and ways of life in addition to hereditary factors [48]. Because of the existence (or lack) of basal cells, which are recognized by particular antibodies paired with racemase activity in luminal epithelial cells, IHC is crucial for verifying the diagnosis of borderline cases of prostate cancer in patients.

One of the most extensively studied biomarkers linked to prostate cancer is p53 (tumor protein p53), which regulates angiogenesis, apoptosis, and the multiplication of cells [49]. Individuals with prostate carcinomas who were immunoreactive to p53 had considerably shorter progression-free intervals and were more likely to experience recurrence. According to research, 21% of prostate tumors tested positive for immunoreactivity, suggesting that p53 tumor suppression gene mutations might contribute to the advancement of some prostate malignancies [50]. An aggressive fraction of prostate cancer is identified by p53 reactivity, which also seems to be a useful independent prognostic marker for low- to intermediate-grade tumors. The study emphasizes the significance of comprehending prostate tumor molecular features and how they affect patient outcomes.

Testicular cancer

Over the past 20 years, there has been an upsurge in testicular cancer, which is the most prevalent malignancy in young adult men. A prevalent risk factor for testicular cancer is aging; in fact, research has shown that men between the ages of 15 and 35 are most susceptible to germ cell tumors. Based on histological features, testicular germ cell tumors (TGCTs) often belong to seminoma and non-seminoma subgroups [51]. Approximately 50% of patients with testicular cancer worldwide receive a diagnosis of seminoma, and the remaining half are found to have mixed TGCTs or various non-seminomas.

*TP53* mutations are present in about 50% of human solid tumors, which are rarely seen in TGCTs but are generally associated with disease activity and a poor prognosis. Clinical correlations were not used in previous studies, even though IHC in adult TGCT revealed positive p53 expression [52]. Combining gene sequencing with IHC analysis revealed that most samples had modest levels of p53 protein expression, with non-seminomas displaying the highest levels and seminomas the lowest [53].

Penile cancer

For every 100,000 men, there are 0.1-1 instances of penile cancer in high-income nations [54]. Penile squamous cell carcinomas (PSCCs) account for 95% of penile cancer cases and are the most common type [55]. The estimated annual incidence of penile cancer is only 26,000 instances, or 1% of all new cases of cancer in men [56]. Preventive measures such as routine neonatal circumcision or risk factors, including smoking, living in an unsanitary environment, and being infected with the human papillomavirus (HPV), can significantly vary the incidence among populations worldwide [57].

With an elevated rate of recurrent tumor suppressor gene alterations, *TP53* was the most frequently changed gene in PSCCs. Like other squamous cell carcinomas (SCCs), PSCC exhibits a mostly mutually exclusive pattern of HPV infection and TP53 mutation [58]. However, the pathophysiology behind the development of HPV-negative PSCCs remains unclear, although it is thought to be connected to mutations in other tumor suppressor genes as well as the tumor suppressor gene *p53* [59]. A subset of individuals with different disease courses may be described by alterations in the *TP53* gene, which could occur later in the development of male reproductive cancers [60]. Positive p53 staining was also seen in 100% of HPV-infected cells [61]. On the other hand, data on p53 expression and HPV DNA presence in penile cancer is inconsistent [62].

Cervical cancer

Worldwide, cervical cancer claimed the lives of 341,831 women in 2020, out of an estimated 604,127 cases. A rise in screening, which can identify cervical abnormalities before they become malignant, contributed to a more than 50% decrease in the incidence of cervical cancer between the mid-1970s and the mid-2000s. Incidence and fatality rates decreased by 11% and less than 1% annually, respectively, among women between the ages of 20 and 24 [63]. HPV vaccine is probably accountable for this. Most cases of cervical cancer are found in people between the ages of 35 and 44. When cervical cancer first appears, it generally shows no symptoms and can only be identified by a pelvic examination or routine Pap screening.

Numerous cancerous growths have been found to have p53 dysfunction, most notably HPV-related cervical carcinoma. The viral protein E6 breaks down the p53 protein, which is one of the ways that HPV causes oncogenesis. The three main pathways of p53 dysfunction, loss of heterozygosity, somatic point mutation, and degradation by HPV’s E6 oncoprotein which targets wild-type p53, are crucial for the development of cervical cancer. The exception in cancer genetics is that *p53* is rarely mutated in early cervical tumors [64].

In HPV-positive cervical condyloma, there is a significant expression of p53. Localized in the nucleus, the gene expression was found to be positive in 47. 3% (96/203) of the cases that were studied [65]. Cervical intraepithelial neoplasia and cervical cancer progress the expression of p53. As a result, while the lack of p53 may not always rule out the possibility of neoplasia, the presence of p53 immunoreactivity can help diagnose a neoplastic lesion.

Ovarian cancer

The NCI report states that over 240,000 new instances of ovarian cancer were reported in women globally, ranking it as the seventh most frequent cancer among females. Ovarian cancer is sometimes referred to as the silent killer because of its often-invasive signs, which make it difficult to treat effectively until the disease is advanced. The stage of the disease at the time of diagnosis directly affects the prognosis for women with ovarian cancer [66]. With the greatest death rate among gynecological tumors, ovarian cancer ranks seventh in terms of frequency among female cancers. To identify individuals who, have ovarian cancer and to distinguish it from benign conditions, cancer antigen 125 (CA125) has been used in the general population [67].

A mutation in *TP53* is the most frequently occurring genetic change associated with epithelial ovarian cancer (EOC) [68]. A malfunctioning p53 pathway is present in nearly all cases of high-grade serous ovarian cancer (HGSOC), with just a tiny percentage of cases having an intact route [69]. Null mutations are more common in the advanced stages of the disease, while missense mutations in *TP53* have been demonstrated to be the most common in ovarian cancers [70]. Hereditary gene mutations are estimated to be the cause of about 10% of epithelial ovarian malignancies; in 97% of instances of HGSOC, pathogenic *TP53* mutations have been found [71]. A study focusing on exons 4-10 of the *p53* gene, an area with almost 95% of the gene cluster’s variations, suggests that peritoneal fluid samples may be useful for the initial identification of *TP53* mutations in EOC [72].

More than 60% of cases of EOC are detected after distant metastases, which contributes to the high death rate. In fact, 90% of women who died from serous EOC had the disease in an advanced stage. In metastasis, the 5- and 10-year survival rates for EOC patients fall to less than 30% and less than 15%, respectively. Overall, 90% of patients with EOC may survive if the illness is identified early. For initial-stage HGSOC, it is essential to deploy multimodal screening techniques that enhance precision and sensitivity [73]. Screening for ovarian cancer is generally initiated with transvaginal ultrasound and CA125 [74].

By using ELISA, it was found that blood antibodies against p53 were detected with 92% specificity in 42% of patients with ovarian serous adenocarcinoma. A molecular test based on the *TP53* mutation could resolve the difficulties associated with HGSOC detection in the beginning. Patients with limited-stage ovarian cancer and those with late-stage ovarian cancer had sera containing p53 autoantibodies (p53-AAb) in between 6% and 7% of cases, respectively. It has been observed that both stage I and stage II serous ovarian tumors have p53-AAb. A potentially effective biomarker for screening is the presence of p53-AAbs in a patient’s circulation, which may be a signal for early diagnosis of EOC [75].

Endometrial cancer

The most prevalent and fastest-growing gynecological malignancy in affluent countries is endometrial cancer. It is a tumor that starts in the endometrium. As the endometrium, or lining of the uterus, is where over 90% of cases of cancer affecting the uterus are found, the American Cancer Society states that cancer affecting the uterine corpus is frequently alluded to as endometrial cancer. Nearly all cases of endometrial cancer occur in postmenopausal women. Endometrial cancer is often diagnosed at stage I as the illness often presents with symptoms early on.

The *TP53* gene was altered in about 90% of serous endometrial carcinomas, with missense mutations accounting for the majority of these changes [76]. In around 25% of cases of endometrial cancer, p53 dysfunction is identified. This dysfunction is directly linked to *TP53* mutation. The prevalence of p53 expression in endometrial carcinomas across all histological types ranges from 17% to 45%. In particular, type I endometrial cancer has a p53 expression rate of 10-44%, whereas type II endometrial cancer has a high incidence of 30-86% [77]. High p53 expression in type I (less aggressive and does not spread fast to other tissues) and type II (more aggressive and spreads outside of the uterus and requires more intensive treatment) is indicative of a poor prognosis. Moreover, nonsense and frameshift *TP53* mutations produce a null phenotype that eliminates p53 immunoreactivity [78]. In the diagnostic assessment of endometrial carcinomas, p53 IHC has become a valuable alternative that can be relied upon to determine the *TP53* mutation status of tumors. As *TP53* sequencing is not always readily available to pathologists, p53 IHC is often used as a stand-in method. The use of p53 IHC was decided upon due to its expediency, affordability, and ease of use. It is regarded by pathologists as the most significant IHC stain for evaluating endometrial cancers. p53 IHC is, therefore, commonly applied to endometrial cancer samples.

Uterine cancer

Malignant mixed mesodermal tumor or malignant mixed Mullerian tumor are other names for uterine carcinosarcoma (UCS), which is a very rare and aggressive cancer. Although UCS constitutes a mere 5% of all uterine malignancies, it causes more than 16% of mortality attributable to uterine cancer [79]. According to estimates from the American Cancer Society, in 2024, 65,000 new cases are expected to be diagnosed, and roughly 12,000 women will lose their lives to uterine cancer. Among rare diseases, uterine cancer is becoming more deadly; since the mid-2000s, the death rate has increased by 1.7% per year.

Over 50% of the *p53*-suppressed uterine genes exhibited elevated expression in *p53*-mutant uterine cancer tissue. Regarding oncogenic genetic fusion uterine sarcomas, the function of the *TP53* mutation remains unknown. However, six of nine (67%) and 21 of 27 (78%) fusion-negative uterine sarcomas and uterine leiomyosarcomas, respectively, had p53 staining patterns suggestive of mutation [80].

## Conclusions

As a diagnostic biomarker for malignancies of the reproductive system, including endometrial, cervical, and ovarian cancers, the *TP53* gene analysis is very effective. Its potential clinical utility is highlighted by its capacity to identify cancer in its early stages, forecast therapy response, and forecast patient outcomes. p53-AAb can be used as an attractive biomarker because *p53* mutations are found in many cancers. The most important pathologic assessment for different types of cancers could entail p53 IHC staining. More importantly, p53-specific indicators may be very helpful in the diagnosis of cancer in patients who are predisposed to tumors. Research is necessary to increase the accuracy and clinical applicability of *p53* mutations, as their sensitivity and specificity may restrict their diagnostic use. Furthermore, a customized strategy for the application in clinical practice is required due to the variability of *p53* mutations among various cancer types and patients. Future studies and developments in cancer detection and treatment are anticipated given the diagnostic potential of the *p53* gene in reproductive cancer.
